# A Mental Files Approach to Delusional Misidentification

**DOI:** 10.1007/s13164-015-0260-5

**Published:** 2015-04-23

**Authors:** Sam Wilkinson

**Affiliations:** Department of Philosophy, Durham University, 50 Old Elvet, Durham, DH1 3HN UK

## Abstract

I suggest that we can think of delusional misidentification in terms of systematic errors in the management of mental files. I begin by sketching the orthodox “bottom-up” aetiology of delusional misidentification. I suggest that the orthodox aetiology can be given a descriptivist or a singularist interpretation. I present three cases that a descriptivist interpretation needs to account for. I then introduce a singularist approach, one that is based on mental files, and show how it opens the way for different and potentially more plausible accounts of these three cases. I reflect on how this mental files approach can be viewed either as a supplement to the orthodox aetiology, or as suggesting an altogether different aetiology. I end by addressing a concern surrounding the explanatory power of mental files.

## Introduction

In this paper, I explore a mental files approach to delusional misidentification. More specifically, I suggest that we can usefully think of delusional misidentification in terms of systematic errors in the management of files. What causes or underpins this mismanagement then becomes the central issue for debates concerning aetiologies of delusional misidentification.

I proceed as follows. I begin by sketching the orthodox “bottom-up” aetiology of delusional misidentification. I suggest that the orthodox aetiology can be given a descriptivist or a singularist interpretation. I present three cases that a descriptivist interpretation needs to account for. I then introduce a singularist approach, one that is based on mental files, and show how it opens the way for different and potentially more plausible accounts of these three cases. I reflect on how this mental files approach can be viewed either as a supplement to the orthodox aetiology, or as suggesting an altogether different aetiology. I end by addressing a concern surrounding the explanatory power of mental files.

## The Orthodox Aetiology of the Capgras Delusion

The standard case of delusional misidentification (although we will encounter others) is the Capgras delusion, the belief that a loved one has been replaced by an identical-looking impostor.^1^ This is delusional (at least partly) because it is tenaciously held, in spite of the fact that there seems to be no evidence to support it. What is striking is that the subject’s perceptual system seems to be working fine: she’s not blind or agnosic in any way. Furthermore, in cases that occur in the context of brain damage (and aren’t accompanied by schizophrenia diagnoses), the subject displays a remarkable degree of normality outside the delusion’s narrow domain. In this section, I sketch the orthodox aetiology of this delusion, present some well-established variations of it, and then introduce a novel variation, which consists in being a singularist or a descriptivist about human cognition.

### Bottom-Up vs. Top-Down Aetiologies

There are two very different approaches to understanding delusions in general, and the Cagpras delusion in particular. The first approach, which has given rise to so-called “bottom-up” models, views the delusion as grounded in some kind of anomalous experience. The delusional subject experiences the world in a way that partly (and as we shall see, perhaps entirely) explains why they believe what they do. Thus, in the case of the Capgras delusion, something about the way the misidentified person is experienced by the subject explains why the person is misidentified.

The other approach, which we will put to one side for most of the paper (it will resurface at the very end), is the so-called “top-down” model, whereby the delusion is not grounded in experience, but directly caused by brain dysfunction (Eilan 2000; Campbell 2001). As John Campbell puts it, ““delusion” is a matter of top-down disturbance in some fundamental beliefs of the subject which may consequently affect experiences and actions” (2001, p. 89). Thus any subjective report, for example, that the Capgras patient’s mother feels unfamiliar, is a consequence of (or an accompaniment to) the delusional belief, but not grounds for it.

Top-down models are definitely a minority.^2^ When I refer to the “orthodoxy aetiology”, I am referring to the whole family of bottom-up models. Bottom-up models come in a number of varieties. These varieties are the result of different views concerning, first, the nature of the experience on which the belief is grounded, and, second, the nature of the process of belief formation, maintenance or evaluation. Prior to presenting these varieties, it is important to present the coarse-grained aetiology on which the more precise varieties are built. I refer to this as the “classic” bottom-up account.

### The Classic Bottom-Up Account

The classic bottom-up account, on which the current orthodoxy is built, is undoubtedly Ellis and Young’s influential (1990) proposal that the Capgras delusion can be understood as a sort of “inverse prosopagnosia”.

People with prosopagnosia have difficulty in the overt recognition of faces. If you show them a picture of a familiar face, they will not be able to tell you whose face it is. And yet, some of them appear to have differential autonomic responses (roughly, affective/emotional responses) to these faces, as measured by heightened skin conductance response (SCR). In other words, although they themselves cannot tell you whose face they are looking at, their affective system seems at the very least to be able to ‘tell’ that it is someone familiar. Borrowing Bauer’s (1984) model for facial processing, whereby there are two streams for processing facial information – one covert, affective and anatomically dorsal, the other overt, semantic and anatomically ventral – Ellis and Young hypothesized that, whereas with prosopagnosia the affective stream for “covert recognition” is intact and the semantic stream for “overt recognition” is impaired, with the Capgras delusion it is the other way around.

This means that the Capgras patient is presented with someone who, thanks to intact semantic processing, looks to them exactly like a loved one, but there is a lack of affective response. The perceived person feels unfamiliar and the patient therefore concludes that this person cannot be the loved one in question.^3^

### Explanationist vs. Endorsement Accounts

There is a debate within bottom-up theories about what *precisely* the nature of the anomalous experience, which is driving the delusional judgement, really is. To borrow Bayne and Pacherie’s (2004) terminology, “explanationist” accounts (e.g., Ellis and Young 1990; Maher 1974) claim that the content of the Capgras patient’s experience is something *sparse* like, “This man feels unfamiliar”, and that the delusional judgment *explains* the bizarre experience. The delusional inference takes something like the following abductive form (Ellis and Young, themselves, call this a ‘rationalization strategy’):

1. This man looks like my father.
2. This man feels unfamiliar (hence doesn’t feel like my father).
  [How do I explain that, although this man looks like my father (and hence should feel like my father), he fails to feel like my father?]
3. He must not *be* my father.^4^

The opposing accounts, so-called “endorsement” accounts (e.g., Bayne and Pacherie 2004) claim that the delusional content is encoded directly in the unusual experience, and all that suffices is endorsement of that content. The content of the Capgras patient’s experience, on such a view, is something *rich* like, “This man is not my father”.

Regardless of the plausibility of either account, it is worth noting an explanatory trade-off. As Pacherie (2009) points out, the explanationist account can more easily explain how the experience gets its content, since the content is so sparse. It can simply claim that there is a disruption in emotional or affective processing. However, it is a bigger explanatory step from the sparse content of the experience to the rich content of the delusion. One *prima facie* problem with this is that, if the experience is sparse and non-specific, why is there not a wider array of potential hypotheses used to explain it? (“Maybe I don’t like mum anymore”, “Maybe I’m tired” etc.). In contrast, the endorsement theorist can get from the experience to the judgement just fine, since they have the same content. However, as Pacherie puts it, where “the endorsement account would appear to be weakest is in explaining how delusional patients could have the experiences that the account says they do” (Pacherie 2009, p.107). We will return to this issue later.

### One-Factor vs. Two-Factor Accounts

The delusion is not just formed briefly but maintained over time and in the face of much contrary evidence, namely, the testimony of others (including people of authority such as doctors) and, of course, the obvious implausibility of the delusional scenario.^5^ Is the affectively anomalous experience enough to account for the delusion? Some (e.g., Maher 1974; Reimer 2009) say that it is. These are called one-factor accounts. Others claim that there needs to be something else to explain the tenacity of the delusion. In addition to the unusual experience, there must be a reasoning bias or deficit (Stone and Young 1997; Davies et al. 2001; Coltheart 2005; Coltheart et al. 2010). These are called two-factor accounts.

Two-factor accounts are probably the more popular of the two (they are widely accepted by other theorists, who are not key proponents of the view, e.g., Bayne and Pacherie 2004; Bortolotti 2009). However, there is something unsatisfactory about both sides of the debate. While one-factor accounts struggle to explain why the delusion is so tenacious, two-factor accounts struggle to give tangible accounts of the second factor. One problem for two-factor theories is that if the second factor is a reasoning bias, then one would expect it to be domain general. This seems to be in conflict with the fact that several delusional patients are strikingly normal beyond the delusion’s narrow domain.

Insofar as both one-factor and two-factor accounts are bottom-up accounts, they are sub-varieties of the orthodox aetiology.^6^

### Descriptivist and Singularist Versions of the Orthodox Aetiology

Now I’d like to draw attention to two other varieties, within the orthodox aetiology, that have thus far gone unnoticed. These varieties stem from two opposing and fundamental positions about how human cognition links up with the world, namely, whether human cognition is property-based or individual-based.^7^ We can think of individual-based and property-based cognition in engineering terms. You could build a robot whose basic cognitive relation to the world is one of qualitative “recognition”. It would go around detecting colours, shapes or patterns, namely, *properties* that can be multiply instantiated. Then you might want to give it a capacity for building “conceptions” of individuals purely out of the properties it can detect. Alternatively, you could build a robot whose basic cognitive relation to the world is one of “tracking”, namely, of keeping track of individual things, independently of, and prior to, the attribution of any property. Saying that human cognition is individual-based is making the empirical claim that human beings are, in an important sense, more like the latter than the former kind of robot. They have a pre-predicative tracking mechanism.

There is an analogy that can be drawn between property-based and individual-based cognitive systems, and a distinction among (mental and linguistic) theories of reference between descriptivism and singularism. Descriptivism, as Recanati (2012) puts it, is the view that “Objects are given to us only qua instantiators of whatever properties we take them to have” (p.3). According to singularism, on the other hand: “Objects are given to us directly, in experience, and we do not necessarily think of them as the bearers of such and such properties (even though the properties of objects are revealed to us when we encounter them in experience)” (p.4). Although the analogy is not perfect, I will, for ease, use “descriptivism” to refer to the position that maintains that human cognition is property-based, and “singularism” for its individual-based counterpart.

Now, what has this got to do with the orthodox aetiology of delusional misidentification? The key lies in the role that singularists and descriptivists can allow the diminished affect to play in identification (and hence misidentification). For the descriptivist, the familiarity of the perceived individual is a property that, in principle, other individuals could also have. A descriptivist version of the orthodox aetiology would look like this. Recall that, according to descriptivism, “objects are given to us only qua instantiators of whatever properties we take them to have” (Recanati 2012, p.3). The subject, in the Capgras case, takes person S to have (among other properties), A (e.g., what the person looks like) and B (e.g., what the person feels like). This person has A, but not B, and therefore cannot be S.^8^ In other words, there are two properties that compete in a rational, evidential calculus.

A singularist version of the orthodox aetiology could look very different. A singularist certainly wouldn’t want to deny that we often judge the identity of something on the basis of its properties. However, that is not what the judgement of identity consists in. This frees up the possibility that there can be other paths to identification. In particular, such a view could allow that “x looks like S” is a judgement about something having a property, whereas “x is S” is a judgement based on a “direct relation” between the subject and the encountered individual, who is “not necessarily [thought of] as the bearer of such and such properties”. One upshot of this is that “x looks like S” and “x is S” (or, more relevantly, “x isn’t S”) won’t compete in a rational, evidential calculus. We will see more on this, and its implications for understanding delusional misidentification.

### Three Cases

The descriptivist version of the orthodox aetiology of delusional misidentification works very well, in principle, for the “standard” cases of the Capgras delusion, which involve a stable (rather than reduplicative) misidentification of a small set of loved ones. It enables us to understand the subject’s judgement, enables us to put ourselves in the subject’s shoes, so to speak (we can ask ourselves “What would it be like if I saw my parents but they felt unfamiliar?”). Furthermore it explains why, in the standard cases, the delusion only concerns loved ones. The subject only misidentifies loved ones because it’s only with loved ones that a feeling of warmth or familiarity is *expected* (namely, it is only loved ones who are taken to have the property of being able to arouse in the subject the relevant sense of familiarity). However, it encounters some, perhaps not insurmountable, obstacles when explaining other cases that one would hope to explain in the same way. I run through them now, and rehearse the responses that are available to the descriptivist. Responses are available, but they are not as neat as those available to the singularist. Indeed some of the responses might even be gesturing in a singularist direction.

#### Case 1: Global Capgras

Many Capgras patients misidentify far more than their loved ones. Ellis et al. (1997) mention a patient who was under the impression that almost everybody in a town had been replaced. The expectation of a feeling of familiarity might not plausibly account for these cases since you don’t expect to feel familiarity when you meet a mere acquaintance.

In response, what could be claimed is that *some* affective response is implicated in identifying everyone that one has previously encountered, even those one doesn’t know very well. Perhaps the worse the affective damage, the larger the set of misidentified people. Perhaps one might even hypothesise that the incremental damage to affective processing would correlate with incremental misidentification, starting with the most familiar, and working towards the least familiar.

#### Case 2: Reduplicative Capgras

The descriptivist struggles to explain the cases where the duplicates themselves are duplicated (i.e., the impostors are replaced, as in the original case described by Capgras and Reboul-Lachaux (1923), where Mme M. reduplicated her husband an estimated 80 times). This cannot be the result of expected emotional response, since the subjects would not expect a feeling of familiarity from the impostor. Indeed, on this view, it is this lack of feeling of familiarity that makes them judge it to be an impostor in the first place.

In response, what could be claimed is that a feeling of familiarity can (and does (cf. e.g., Hirstein and Ramachandran 1997)) grow for “the impostor” and becomes constitutive of the subject’s conception of them. But then, if this feeling were to suddenly wane, then the now-familiar impostor might themselves be judged to be an impostor.

#### Case 3: The Frégoli Delusion

A patient with the Frégoli delusion will claim that a known individual has taken on the appearance of a nearby stranger. In other words, it involves claiming that someone is S in spite of looking nothing like S.^9^ The descriptivist aetiology, since it explains the lack of identification in terms of diminished affective response, would presumably explain the Frégoli delusion in terms of heightened affective response (as suggested by Davies et al. 2001). This works for the Capgras delusion because the relevant candidate identity is suggested to the subject by the encountered individual’s appearance, and that is defeated by a lack of warmth (or sense of familiarity). In order for it to work with the Frégoli delusion, one needs to make the claim that affect alone can pick out a specific identity. But this is perhaps implausible. One might expect a heightened sense of warmth or familiarity to elicit a vague “Haven’t I met you before?” sort of response.

In response, one can take an explanationist or an endorsement angle. One can (adopting an explanationist strategy) claim that the experience of heightened familiarity is indeed vague, but a more precise hypothesis is adopted in order to explain it. Of course, then we need an account of why that hypothesis is adopted and not another. Alternatively, (adopting an endorsement strategy) one can view the *experience* as precise. One might say that, although we might not be consciously aware of this, we do have fine-grained affective profiles for specific individuals. This is perhaps not too implausible. However, as we will see, it is going somewhat in the direction of singularism.

## A Mental Files Approach

In this section I present a singularist version of the orthodox aetiology and, in particular, one that uses the notion of mental files. The approach suggests that we think of delusional misidentification in terms of a mismanagement of mental files. Afterwards, however, we will consider whether a mental files approach might be viewed as going beyond the orthodox bottom-up aetiology altogether.

### Identification and Predication

Assuming individual-based cognition, what happens when you judge that someone you perceive is a certain individual is something that is importantly, both logically and psychologically, very different from what happens when you judge that someone has a certain property or appearance (e.g., red hair). In the latter case, you are predicating a property of an individual. It is a judgement of the form *Fa*. You might have quite a rich conception of that individual, know them rather well, or you might only know the bare minimum to achieve some kind of demonstrative mental reference (“This stranger, walking past me now, has red hair”). In contrast, identifying (or re-identifying) someone involves the judgement that an individual currently perceived (or otherwise relevant) is the very same individual as another individual, one you have previously encountered or been somehow familiarised with (e.g., someone you have read about or seen on TV), in principle independently of any properties they may have. It is of the form *a=b*, not *Fa*.

Although grammatically speaking, ‘=’ can be treated as a two-place predicate, psychologically speaking, if cognition is individual-based, the mental act of identifying is not the mental act of predicating. When you judge that this individual (person, dog, set of keys) is the same as one encountered in the past, this does not consist in the attribution of any specific property (although it may, of course, entail that some very general properties have to be attributed, e.g., the property of being a ‘thing’). At a very minimum, the individual identified has to have some kind of informational salience *as an individual* (as opposed to *as a bearer of certain properties*).^10^ You cannot identify an individual that you have not encountered in *any way* before.^11^ Suppose we are walking down the street and I point to a complete stranger and I ask you: “Who is that?” I am asking you to identify that individual, but this is an impossible task. I am asking you to draw a connection of identity between the encountered individual and a previously encountered individual, but where there is no previous encounter.

The fact that identification does not consist in attributing a property is in line with a rather basic truism: *a* at t1 and *b* at t2 can be the same individual in spite of having different properties (i.e., persistence through change), and *a* and *b* can be distinct individuals in spite of having the same properties. This truism has certain epistemic consequences: you can be correct in judging that *a* at t1 and *b* at t2 are the same individual in spite of having very different properties, and that *a* and *b* are distinct individuals in spite of having the same properties.^12^

As we will see, mental files can be a useful way of thinking about this difference between identification and predication. While identification involves merging (or linking) two files, predication involves putting information into a file.

### Introducing Mental Files

Talk of mental files provides us with a helpful way of thinking about singularist psychosemantics. Mental files are opened upon an encounter with an individual and then filled with information that is taken to pertain to the individual.^13^ Then when that individual is re-encountered, that file is retrieved, and more information can be put in the file. The content of a file for an individual constitutes the subject’s conception of that individual. This conception will involve a variety of different kinds of information that is taken to pertain to that individual; what they have done, when the subject has encountered them in the past, character traits etc., as well as what they look like. This conception can be false in many ways, and yet still be about that individual, since it is the initial encounter that caused the opening of the file that determines the individual that the file is about. Thus, mental files are “non-descriptive modes of presentation”, or to put it in terms that evoke less of a Neo-Fregean framework, they are non-descriptive singular mental representations. Their correctness conditions do not evoke properties, but specific individuals. Thus, correct retrieval of a mental file occurs when it is retrieved for the *very same* individual that the file was created for, regardless of how much their detectable properties may have changed. Retrieval of a file is incorrect if it is retrieved for an individual for whom it was not created, (regardless of how much that individual shares detectable properties with the initial individual). Alternatively, an error occurs if a new file is created when an old file should have been retrieved. This is the mistake that is relevant for the Capgras delusion.

One important use for mental files is in modelling discoveries about numerical identity or distinctness. The idea is that when you discover that individuals encountered on two different occasions (or in different ways) are in fact one and the same individual, you merge two files into one.^14^ Conversely, if you thought that two individuals were identical, but then realised that they are in fact distinct, then your file divides into two files. To illustrate both merging and division in one unlikely example, suppose you start working in a new research centre. After a few weeks of walking past new colleagues in the corridor and politely saying “Hello”, but prior to the official meet-and-greet, you are under the impression that, among your new colleagues, there is one bearded man and two women with glasses. However, at the meet-and-greet, you realise that there are in fact two bearded men in your centre, and you had in fact encountered both, but hadn’t realised that they were distinct individuals. Here your file for “the bearded guy at the office” splits into two. Strikingly, you furthermore realise that there is in fact only one woman with glasses, you just thought that there were two (because she sometimes wears her hair up). There your two files merge into one.

Now, there are (at least) two importantly different kinds of files: “Demonstrative” and “Stable” files. When you encounter an individual for the first time, you open a file. This file is dependent on the current perceptual context. It is a file for, e.g., “this man I’m perceiving now”. This is a *perceptual*-demonstrative file. However, when the man in question is no longer perceived, you may still be able to think of him in virtue of episodic memory, for example, by recollecting the context of the encounter. This becomes a *memory*-demonstrative file. It is a file for “the man I was perceiving then”. However, when you know enough about a person, and have a stable enough conception of them (and in particular, attaching a proper name to them can be a useful and cognitively low-cost way of doing this (see Jeshion 2009)), you have a “stable” file for that person. You can think about them in any context: you neither have to perceive them, nor do you have to remember any specific encounter. But what happens when you *perceptually* encounter someone that you have a stable file for? What happens is that you open a perceptual-demonstrative file, retrieve your stable file, and merge (or link) the two. This amounts to judging “this man here present, is the very same as this man of whom I have stable conception”. If the individual for whom you opened the perceptual-demonstrative file is not the individual for whom you initially opened the stable file, then this is one kind of misidentification. This is mistaking somebody for somebody else. Another kind of misidentification is failing retrieve any stable file, when doing so would have been appropriate. This would involve treating somebody as a stranger when they are in fact not a stranger.

### (Mis)identification as (Mis)management of Files

In a paper that puts forward a ‘theory of integrated tracking’ Bullot (2009) presents the example of Mary and her spouse George. Mary successfully tracks George, ‘first as briefly located in her left visual field at t1, then as a voice saying “goodbye” at t2, and eventually as somebody who has arrived back after a day at work and can be perceived in a multimodal experience at t3’ (Bullot 2009, p.357).^15^ We can think of this in terms of mental files. At t1, t2 and t3, Mary opens a perceptual demonstrative file and merges it with her successfully retrieved George file, thereby correctly identifying him throughout. Let’s suppose, instead, that Mary is brain-damaged between t2 and t3, and asserts, to George’s horror, on his return: “You’re not George!” What is happening here is that at t3, Mary has failed to retrieve her George file, and instead has opened a new demonstrative file, for “This man here present”, which does not merge with the George file.

There is an important question about what has caused the failure to retrieve the file. And clearly, it differs from a misidentification on the basis of, e.g., prosopagnosia, or amnesia. Mary, if she is like the Capgras patients described in the clinical literature (e.g., Hirstein and Ramachandran 1997, Lucchelli and Spinnler 2007), can process the appearance of faces (not prosopagnosic) and remembers all of the relevant encounters (not amnesic). One might even hypothesise that all the information is *there*, it is just being *mismanaged*.

Now, it may well be that file-retrieval is triggered by something in affective processing. Alternatively, it could be (in the spirit of a top-down aetiology) that an affective response is a result, and not a cause, of file retrieval and that this has been directly damaged by the lesion. These are hypotheses that might be testable somewhere down the line, and I have no intention of adjudicating between them here. The important point for our purposes is that the misidentification need not be based on an inference on the basis of properties that the perceived individual is taken to have or not have (including the property of “feeling a certain way” to the subject).

### Fleshing out the Endorsement Account

Recall the explanatory trade-off between the explanationist and the endorsement accounts. The challenge for the endorsement account is to explain how it is that the experience can have the rich misidentificational content that the account claims it has. One can view the contribution of the mental files approach as a way of addressing this. Two things should be said at this point.

First, this would suggest that identity can enter into perceptual content. In other words, you can perceive (or fail to perceive) a person’s identity directly, without inferring it. It would be interesting to see how this idea relates to recent debates about the kinds of *properties* that enter into the content of perceptual experiences. For example, Susanna Siegel (2006) has recently argued that kind-properties can enter into perceptual content. Is the claim that identity enters into perceptual content a stronger or a weaker claim than this? Answering this will in turn depend on whether one is a singularist or a descriptivist. If one is a singularist in general, and a files theorist in particular, then it is arguably a weaker claim. There’s nothing mysteriously rich about the content of the Capgras experience according to the endorsement model. That is because taking someone to be a certain individual is something primitive in human cognition; it is not, as the descriptivist would have it, something that is built up out of more fundamental properties.

The second point is to do with how this relates to the one-factor vs. two-factor debate. It seems that, even if the content of the delusion is already in the perceptual experience, this still doesn’t explain why the delusion is so tenacious. After all, we often override what our perceptual states tell us. For example, with the Muller-Lyer illusion, we don’t go on believing that the lines are different lengths just because they still appear to be so. Thus it seems that endorsement models, as fleshed out by mental files, would need a second factor as much (or perhaps almost as much) as the explanationist model. This is something that endorsement theorist seem happy to embrace (Bayne and Pacherie 2004).

## Accounting for the Cases

Here I will present the kinds of accounts of the cases that are available to a singularist, files-based, version of the orthodox aetiology. Such an aetiology can be thought of as an endorsement account that has been fleshed out with mental files.

### Case 1: Global Capgras

Recall that the descriptivist needs to explain Global Capgras in terms of how mere acquaintances, and not just loved ones, would be expected to arouse at least some sense of familiarity in the subject. In contrast, the mental files approach wouldn’t rely on such affective expectations on the part of the subject. Global Capgras would be explained in terms of a global failure to retrieve files, and therefore of erroneously opening new demonstrative files for people who have in fact already been encountered. Even though the subject *remembers* all the previous encounters with those people, he doesn’t remember them as encounters with *those very individuals*, but rather with different, yet identical-looking, individuals.

Much more work would be needed, but the singularist could then hope to flesh out the empirical details of her account, perhaps by hypothesising that the file retrieval is caused, or constituted, by some kind of “affective processing”.^16^ Note, as we said before, that the singularist would not take the damage to affective processing to give rise to an experience like “This person doesn’t feel like S”, which would evidentially compete with the encountered person’s overt appearance. Rather, what it is to directly judge that someone is or is not a certain known individual is (at least partly) caused or underpinned (where “caused” would imply asynchrony, and “underpinned” simultaneity) by something that gets experimentally detected as “affective processing”.

### Case 2: Reduplicative Capgras

The descriptivist has to explain why the impostor himself would be replaced by another double in terms to “the impostor” becoming familiar, and then the relevant affect in turn being disrupted or diminished. In contrast, the mental files approach would view this as an excessive opening of new files, and failure to retrieve old ones. Pushed to extremes, a total mismanagement would mean that every encounter with an individual would be as if that individual was being encountered for the first time; every discrete encounter would result in further reduplication. Again, more work would need to be done, but the singularist could view the mismanagement as being caused by disruption to low-level cues that enable file-retrieval. Perhaps these could count as “affective” or as involving “affective processing”.

### Case 3: The Frégoli delusion

As we said, in order for the descriptivist to account for the Frégoli delusion, she needs to make the claim that a feeling of familiarity can pick out a specific individual. In contrast, given the mental files approach, one would expect a specific individual to be selected, since it would involve hyper-active file retrieval (which may be caused, accompanied, or underpinned by something affective). It would involve retrieving a file when it is inappropriate to do so; the excessive drawing of connections of identity between currently perceived individuals and individuals encountered in the subject’s past. There would be nothing vague, or gut-feeling-like, about it. This seems to be reflected in the assured assertions of Frégoli patients. These assertions do not seem to be inferentially arrived at on the basis of a vague feeling: the person’s identity is asserted with certainty.

## An Alternative Contribution of Mental Files?

We have seen that a potential contribution of mental files is within an orthodox, bottom-up aetiology. In particular, it can be viewed as providing theoretical support and motivation for the idea, central to an endorsement model, that experience can be of specific individuals. However, one could alternatively view mental files as suggesting something rather different, something more akin to a top-down model (and which, like all top-down accounts, bypasses the fraught one-factor vs. two-factor debate). As we have seen, endorsement accounts can be both singularist, and committed to an orthodox, bottom-up, aetiology. Is it possible, let alone plausible, to keep a commitment to the former, while discarding the latter? Perhaps it is.

Endorsement accounts show an implicit adherence to what we might call a “testimonial view of perceptual experience”. This view takes perceptual experience to be (always) epistemically analogous to somebody telling you something, in the sense that you can weigh up whether or not you should trust it. Such “weighing up” might, for example, be done on the grounds of belief about the reliability of the source. If you know you have just taken a strong hallucinogen (or been informed that you have brain damage), you might have reason to distrust your senses, much like how you might distrust an informant you knew to be untrustworthy. Alternatively, you can distrust your senses because they are presenting you with something which doesn’t square with lots of other information, much like you can distrust an informant who, for all you know, is generally reliable, but who tells you something deeply implausible. Of course, the Capgras patient has both of these reasons to distrust what her perceptual experience is telling her. Explaining why the patient goes against these reasons is what the two-factor theory is trying to achieve.

However, suppose that perceptual experience isn’t always something that presents you with a content that you can weigh up for plausibility, and always in principle reject. Suppose that sometimes it *forces* you to judge something. Suppose that this is what happens in the Capgras case. Whatever mechanism is responsible for the mismanagement of files doesn’t just present you with a content that you can consider: it gives rise to an experience that carries inbuilt doxastic weight. We earlier compared the Capgras experience with the Muller-Lyer illusion as a way of showing that the endorsement account also needs a second factor. Humans, we claimed, can, as in the Muller-Lyer illusion, form beliefs that go against what their perceptual experience is telling them. However, perhaps this comparison is a poor one. Perhaps the obvious differences between the Muller-Lyer Illusion and the Capgras experience provide important clues. The Muller-Lyer Illusion, and other illusions, concern judgements of *appearance*, not judgements of identity. Properties such as length, for example, are multiply instantiable.

For a singularist, a judgement about the length of a line is predicative in a way that a judgement about identity isn’t. Your perceptual experience can encourage you to predicate one thing, but you can override it with competing indirect evidence (such as measuring the lines with a ruler). But in cases where the judgement is not predicative, but one of identity being drawn between two individuals, this might not be the right kind of thing that competing perceptual evidence can *directly* encourage or discourage. Compare two attempts at getting someone to revise their beliefs. In one case, the belief is predicative, in the other it is not.

- A1 – “Those lines are different lengths because they look different lengths.”
- A2 – “I assure you they are the same length: go and measure them!”
- B1 – “That’s not my father” (where “my father” is a directly referring singular term).
- B2 – “Of course it is your father. It looks exactly like him!”

The problem with B2, according to the mental files theorist, is that it wrongly assumes that the judgement of identity (or lack thereof) is grounded in properties. But perhaps B1 is not grounded in properties. If this is the case, then B2 is bound to be wide of the mark, and unconvincing to the delusional subject. The predicative judgement “This person looks like my father” is one that the delusional subject has already made; B2 is simply restating it, and, in any case, it simply doesn’t evidentially compete with B1, which has been caused by an inability to retrieve the correct file.

To sum up, then, perhaps these tracking deficits can, because tracking is so primitive, produce experiences that are somehow *indubitable*. Or, to put it more clearly, perhaps these deficits produce judgements that are somehow immune to revision in the light of other evidence that is overtly present in the experience (e.g., what the individual looks like). Indeed, one might even think that, for explaining the delusional state, one shouldn’t talk in terms of inferences on the basis of experiential evidence, but rather explain it at the neural level. Perhaps by the time that we get to conscious experience the misidentification is already there. And here we seem to be considering something rather similar to the top-down theories we put aside at the start. Then, of course, if this is the case, then the entire one-factor/two-factor debate is misguided.

I do not want to support either the bottom-up, fleshed-out endorsement account, or the top-down account. I do think, however, that it is important to consider the latter as an option.

## A Concern: Explanation or Re-description?

To finish, I’d like to address a very general concern that someone might have about the mental files approach. Is this talk of files anything more than just a fancy re-description? My answer to this is that, in an important sense, it is a re-description. However, it is a re-description that carries tremendous explanatory potential. It provides us with an easy and intuitive way of talking and thinking about the dynamics of singular cognition. In particular, it does so with the crucial distinction between a file and its content. This is metaphorical insofar as there are not *literally* files, and they do not literally *contain* things. However, there are so many phenomena that can be thought of in precisely this way, namely, in terms of merging, splitting, opening and adding to files. For our purposes, the functional distinction between a file and its content nicely captures the fact that we can take an individual to be a certain individual, independently of the properties that we took them to have. This rather profound yet simple point, which is at the heart of singularist psychosemantics, is crucial in helping us understand delusional misidentification. The notion that someone could judge that someone is not S in spite of looking just like what the subject took S to look like (Capgras) or that someone could judge that someone is S in spite of looking nothing like them (Fregoli) is much less outlandish to the singularist than to the descriptivist.^17^

## Footnotes

1 There is a debate concerning whether delusions are beliefs. This debate doesn’t concern us (at least not directly), so, for ease, I will assume that delusions are beliefs.

2 Top-down views have found little support, perhaps, in part, because theorists feel it’s really not clear where to start looking, as a cognitive and brain scientist, for direct neural causes of beliefs. It is much easier to hypothesise about how certain kinds of brain damage might affect the subject’s experience, and then we might get some idea, at the personal level, of how the experience gives rise to the belief.

3 This model was given experimental support (Hirstein and Ramachandran 1997, Ellis et al. 1997) when it was discovered that, in contra-distinction to prosopagnosia, Capgras patients show diminished SCR when presented with familiar faces. Of course, as top-down theorists are quick to emphasise, this data is compatible with the hypothesis that reduced autonomic response is a consequence, rather than a cause, of the misidentification. In other words, the individual feels unfamiliar because she has been judged to not be the subject’s mother, rather than the other way around.

4 The Ellis and Young aetiology looks like an explanationist model. However, it is not obvious that lack of affective response is something on the basis of which *the person* performs an inference, or if it is something *subpersonal* which presents the subject with the experiential content: “This is not my father”.

5 The patients are sometimes sensitive to this implausibility. Patient DS (Hirstein and Ramachandran 1997, p.438) when asked why this man was pretending to be his father, he replied “That is what is so surprising, doctor; why should anyone want to pretend to be my father? Maybe my father employed him to take care of me … paid him some money so that he could pay my bills.”

6 There is a sense in which a top-down account can be seen as having only one factor (e.g., one deficit). However, given that the one factor vs. two factor debate centres around whether the anomalous experience alone suffices to explain the delusion, top-down account clearly cannot take part in the debate.

7 By “individual”, I mean in the broad sense of being a unique particular. I mean it to include objects of any kind: animals, persons, chairs, billiard balls.

8 A view that doesn’t build on Ellis and Young’s (1990) hypothesis, but which is still implicitly committed to descriptivism is the view of Hirstein and Ramachandran (1997) and Hirstein (2010). They emphasize the difference between the perceivable properties of an individual, and our representations of them “from the inside”. According to them, the Capgras delusion occurs when our “internal” representation of the person is damaged or inaccessible. This produces the impression of someone who looks right on the outside, but seems different on the inside (that is why Hirstein 2010 claims that these misidentification syndromes are mindreading disorders). It is illustrative to see how this is descriptivist. On their view, we represent two different kinds of *properties* of a person: their appearance, and what they are like “as a person.” In contrast, I want to emphasize the central singularist insight that there is a deep distinction between recognizing *properties* and tracking *individuals*. The latter is independent of any property, even the “internal” ones. I can, in principle, see someone as a certain individual, even though both her appearance and her personality have changed beyond all recognition. Hirstein’s view also struggles to account for the delusional misidentification of inanimate objects (e.g., Alexander et al. 1979).

9 Feinberg et al. (1999), for example, report a brain-damaged patient, BJ, who: …approached a severely disabled, wheelchair-bound patient in his early twenties whom he had never met before, and claimed that the patient was his younger son. […] He maintained this misidentification despite clear differences in physical appearance between the falsely identified patient and his son. Even when these distinctions were pointed out by staff, BJ maintained his original assertion that the patient was his son. (1999, p.378)

10 This kind of ‘significance as an individual’ happens less often with inanimate objects, because we only rarely care about the particularity of inanimate objects, we care about their properties (if you break something of mine, unless it has sentimental value, I don’t care if you replace it with an identical duplicate).

11 I mean this in the broad sense of ‘encounter’, to include any kind of informational channel (e.g., including the media). You can clearly meet someone for the first time and say, “It’s great to finally meet you: I’ve heard so much about you”. That counts as identification, but then the “hearing so much about them” counts as “encountering”.

12 Note how the former is the kind of judgement that someone with the Frégoli delusion (the delusion that people from one’s past are taking on the appearance of surrounding strangers) makes, and that the latter is the kind of judgement that someone with the Capgras delusion makes; both, of course, are erroneous.

13 Mental files are used in a number of subtly different ways by different theorists. What I am calling “mental files”, some mental file theorists will call “singular files” since they want to allow that there are mental files that aren’t singular (e.g., files for natural kinds). However, I haven’t yet been convinced by the need to use mental files for anything other than singular cognition, so I am happy to use either term.

14 Some theorists (e.g., Lawlor 2001; Recanati 2012) prefer to think of this in terms of “linking” two files rather than “merging” two into one. Hence they would deny that there are as many files as salient individuals in the subject’s world. Perhaps they would say there are as many files as there are singular modes of presentation, and that subjects can maintain more than one mode of presentation for (someone they know to be) one individual. One motivation behind this may be that it makes it easier to correct a mistaken identification.

15 Bullot explicitly contrast this with the Capgras patient who, ‘may fail to perform the integrative tracking of their spouse due to a delusional belief that the person perceived at t1 has been replaced by an impostor at t3’ (ibid). Although this is broadly correct, it is phrased in a misleading way. It suggests that the tracking has failed as a result of the subject’s delusional belief. However, on the view I am proposing here, this is the wrong way around: the subject’s delusional belief is the result of a failed tracking mechanism. This is not pedantic: it is crucial to what is distinctive about a singularist, tracking-based, view that is grounded in an intuitive perceptual tracking mechanism.

16 A problem that all aetiologies share is that the term “affective processing” is so vague. For example, sometimes it seems to refer to a conscious emotional process, sometimes to a physiological response or low-level neural activation.

17 Indeed a singularist view can even go some way towards explaining what is delusional about delusional misidentification. Delusion attribution (roughly) occurs when a subject’s cognition pulls in the opposite direction to our practices of interpersonal justification. One might then say (and it helps us understand delusional misidentification, both aetiologically, and *qua* delusional phenomenon) that the singularist is right: cognition is individual-based. However, our practices of interpersonal justification, insofar as we cannot *but* point to publicly accessible things in our justificatory explanations, namely, to properties, have a descriptivist flavour. Thus, granting singularism, we would expect misidentification to be the kind of judgement that would elicit attribution of delusional status. Other kinds of judgement (e.g., the ones expressed in the contexts of schizophrenia diagnosis) need to be far more florid in content in order to count as delusional.
